# Male advantage observed for in vitro fertilization mouse embryos exhibiting early cleavage

**DOI:** 10.1002/rmb2.12355

**Published:** 2020-10-30

**Authors:** Yosuke Kawase, Takanori Tachibe, Nobuo Kamada, Kou‐ichi Jishage, Hiroyuki Watanabe, Hiroshi Suzuki

**Affiliations:** ^1^ Chugai Institute for Medical Science, Inc Gotemba Japan; ^2^ Obihiro University of Agriculture and Veterinary Medicine Obihiro Japan

**Keywords:** early cleavage, embryo, male advantage, mice, sex ratio

## Abstract

**Purpose:**

Mouse embryos forming blastocoele early vs those forming late are predominantly male. We examined whether the male advantage could be recognized at an earlier stage of development.

**Methods:**

The IVF embryos were classified into early, intermediate, and late development groups based on the time of the third cleavage, and the *Zfy* and *Xist* genes were detected to identify their sex in the classified embryos. Furthermore, embryos that were classified based on the time of the third cleavage were transferred to recipient animals and the sex ratio of the fetuses was determined at birth.

**Results:**

Approximately 90% of the early‐developing embryos that exhibited third cleavage as early as 47 hours after insemination were male when analyzed using PCR at the blastocyst stage. PCR analysis showed that 61% of the intermediate‐developing embryos (third cleavage occurring 48‐50 hours after insemination) and 45% of late‐developing embryos (third cleavage occurring at 51 hours or later postinsemination) were male. After embryo transfer, the early‐developing embryos produced 80% males, while intermediate‐ and late‐developing embryos produced 56% and 45% males, respectively.

**Conclusions:**

Male embryos tend to develop faster than female embryos during early stage of preimplantation in mice.

## Graphical abstract

Embryos that were classified based on the time of the third cleavage were transferred to recipient animals, and the sex ratio of the fetuses was determined at birth. Approximately 90% of the early‐developing embryos that exhibited third cleavage as early as 47 h after insemination were male when analyzed using PCR at the blastocyst stage. PCR analysis showed that 61% of the intermediate‐developing embryos (third cleavage occurring 48–50 h after insemination) and 45% of late‐developing embryos (third cleavage occurring at 51 h or later postinsemination) were male. These results indicated that male embryos tend to develop faster than female embryos during early stage of preimplantation in mice. In fact, after embryo transfer, the early‐developing embryos produced 80% males, while intermediate‐ and late‐developing embryos produced 56% and 45% males, respectively.

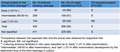

## 1 INTRODUCTION

Sex ratio skews have frequently been reported among mammals.^1^ In mice, it has been reported that mouse embryos forming blastocoele early versus those forming late are predominantly male.^2^, ^3^ Although it has been reported that sex‐determining region genes, *Sry* and *Zfy*, are transcribed as early as the 2‐cell stage in mouse embryos,^4^ a male advantage at earlier stage of embryonic development than blastocyst stage has not been examined yet. It might be possible that the male advantage could be recognized at an earlier stage of development in mice. In this study, in vitro fertilized mouse embryos were classified as early, intermediate, and late development based on the time required for the third cleavage. We analyzed the presence of *Zfy* gene on Y‐chromosome ^5^ and *Xist* gene on X‐chromosome ^6^ at the blastocyst stage to identify the sex of the developing embryo. Furthermore, embryos that were classified at the third cleavage were transferred to recipient animals, and the sex ratio of the fetuses was determined at birth.

## 2 MATERIALS AND METHODS

### 2.1 Animals

Adult C57BL/6J and ICR mice were purchased from CLEA Japan Inc (Tokyo, Japan). The animals were housed in a barrier unit at 24 ± 1°C with relative humidity of 50 ± 10% under a 12 hours light/day cycle (lights on 07:00 to 19:00). They were allowed free access to standard laboratory chow (CE‐2, CLEA Japan) and tap water. All experiments were performed in accordance with the guidelines for the care and use of animals approved by the internal regulations for animal use at Chugai Pharmaceutical Co., Ltd., which have been approved by the Association for Assessment and Accreditation of Laboratory Animal Care International. All animal experimental protocols were approved by the Institutional Animal Ethics Committee, Chugai Pharmaceutical Co., Ltd.

### 2.2 Collection and fertilization of mouse oocytes

In vitro fertilization (IVF) and in vitro culture of mouse embryos were performed using previously described techniques.^7^, ^8^ Oocytes were collected from the ampullary region of the oviducts of superovulated C57BL/6J females 16 hours after human chorionic gonadotropin (hCG) injection. Then, the oocytes were inseminated in TYH medium ^7^ with preincubated C57BL/6J epididymal spermatozoa. Approximately 5‐6 hours after insemination, all the fertilized eggs, at the pronuclear stage, were washed twice and cultured in Whitten's medium ^9^ supplemented with 0.1 mM EDTA ^8^, ^10^ for 96 hours at 37°C in 5% CO_2_ in air. Oocytes with two distinct pronuclei and a second polar body were considered to be fertilized.

Since the third cleavage was observed from 47 hours after insemination, and its peak was 48‐50 hours, the in vitro fertilized embryos were classified as “early” (≤47 h after insemination), “intermediate” (48‐50 hours after insemination), and “late” (≥51 hours after insemination) development based on the time required for the third cleavage. In the second series of experiments, embryos were classified as “early” (≤40 hours after insemination) and “late” (≥41 hours after insemination) development by the time of the second cleavage. Since the second cleavage occurred from 38 to 43 hours after insemination, it was classified into two, up to 40 hours and after 41 hours.

### 2.3 Sex determination of the embryos

The sex of embryos was determined at the blastocyst stage using PCR to identify the Y‐ and X‐chromosome‐specific genes, *Zfy* (Y‐chromosome‐linked zinc finger protein), and *Xist* (X‐inactive specific transcript), respectively. Two sets of oligonucleotide primers for *Zfy* (Forward: 5′‐GAC TAG ACA TGT CTT AAC ATC TGT CC‐3′, Reverse: 5′‐CCT ATT GCA TGG ACA GCA GCT TAT G‐3′) and *Xist* (Forward: 5′‐AGG ATA ATC CTT CAT TAT CGC GC‐3′, Reverse: 5′‐AAA CGA GCA AAC ATG GCT GGA G‐3′) were used. For PCR amplification, 5 μL of PCR buffer (10 × EX Taq buffer; Takara, Shiga, Japan), 0.1 μL of each primer (10 pmol), 8 μL of dNTP mixture (Takara, Shiga, Japan), 0.25 μL of EX Taq polymerase (1.25 units, Hot Start Version, RR006Q, Takara, Shiga, Japan), and 26.55 μL of dH_2_O for PCR (Water deionized & sterilized, 06442‐95, Nacalai Tesque, Kyoto, Japan) were added to the tubes containing each embryo in a final volume of 50 μL of reaction mixture. The samples were heated at 95°C for 5 minutes for denaturation. Then, the samples were amplified in a GeneAmp 9700 PCR system (Perkin Elmer, MA, USA) for 40 cycles: denaturing at 95°C for 1 minute, annealing at 55°C for 1 minute, and extension at 72°C for 1 minute. At the end of the 40 cycles, the samples were kept at 72°C for additional 7 minutes for extension. PCR products (10 μL) were characterized by electrophoresis on 3% agarose gels (Agarose, A6013, Sigma‐Aldrich) and visualized using ethidium bromide staining. When the *Zfy* (183 bp) and *Xist* (234 bp) specific bands corresponding to respective amplified fragments were visible, the embryo was considered male. Embryos positive for *Xist* but negative for *Zfy* were considered females.

### 2.4 Embryo transfer

For the embryo transfer experiment, early‐, intermediate‐, and late‐growing embryos were transferred after the third cleavage into the oviducts of pseudopregnant ICR recipients at 0.5 dpc.^11^ The recipient animals were sacrificed at 19.0 dpc. The sex of the fetuses was determined by visual inspection of the urinary papilla. As a control, the sex of the fetuses derived from natural mating was also determined and the sex ratio was calculated.

### 2.5 Statistical analyses

Statistical analysis of the experimental data was performed using the chi‐square test. Statistical significance was set at *P* < .05.

## 3 RESULTS

As shown in Table 1, sex ratio calculated for each group of embryos indicated a significant deviation toward the males within the early‐developing embryo (90%) and intermediate‐developing embryo groups (61%), while late‐developing embryos did not show significant deviation from the total sex ratio (45%). Altogether, 59% (268/452) of embryos were identified as male. This percentage was significantly higher than the expected ratio. When embryos were not classified time of the third cleavage and were determined sex ratio, 56% (229/411) of embryos was male. This percentage was also significantly higher than the expected ratio. These results indicated a skewed sex ratio of the mouse preimplantation embryos fertilized in vitro.

**Table 1:** Sex ratio of in vitro fertilized mouse embryos determined at the time of third cleavage by PCR analysis

Groups based on the time of third cleavage^b^	No. of embryos showing cleavage	No. (%) of males identified by PCR	Chi‐squared analysis (*P* < .05)^a^
Early (≤47 h)	49	44 (90)	S
Intermediate (48‐50 h)	259	159 (61)	S
Late (≥51 h)	144	65 (45)	NS
Total	452	268 (59)	S
Not classified	411	229 (56)	S

Abbreviations: NS, not significant; S, significant.

^a^ Comparison between the expected ratio and the actual ratio obtained for respective trial.

^b^ Culturing embryos fertilized in vitro were classified as to “early” (≤47 h after insemination), “intermediate” (48‐50 h after insemination), and “late” (≥51 h after insemination) development by the attainment time of the third cleavage in culture.

The results from the third cleavage roused our interest in a male advantage at the earlier cleavage, namely the second cleavage. As shown in Table 2, when embryos were classified as early‐ and late‐developing embryos based on the time of second cleavage, significant deviation toward males was observed in the early‐developing embryos (67%). These results indicated that male embryos tend to develop faster than female embryos during early stage of preimplantation development, in addition to a significant deviation in primary sex ratio in mice. Results of embryo transfer experiment (Table 3) showed that among the fetuses that developed from early‐developing embryos at the time of third cleavage, males were predominant (80%).

**Table 2:** Sex ratio of in vitro fertilized mouse embryos determined at the time of second cleavage by PCR analysis

Groups based on the time of second cleavage^b^	No. of embryos determined	No. (%) of males identified by PCR	Chi‐squared analysis (*P* < .05)^a^
Early (≤40 h)	81	54 (67)	S
Late (≥41 h)	122	69 (57)	NS
Total	203	123 (61)	S

Abbreviations: NS, not significant; S, significant.

^a^ Comparison between the expected ratio and the actual ratio obtained for respective trial.

^b^ Embryos were classified as “early” (≤40 h after insemination) and “late” (≥41 h after insemination) development based on the time of the second cleavage.

**Table 3:** Sex ratio of mouse fetuses derived from IVF embryos classified based on the time of third cleavage

Groups based on the time of third cleavage^b^	No. of embryos transferred	No. (%) of implantation sites	No. (%) of developed to fetuses	No. (%) of males	Chi‐squared analysis (*P* < .05)^a^
Early (≤47 h)	64	61 (95)	40 (63)	32 (80)	S
Intermediate (48‐50 h)	393	304 (77)	153 (39)	85 (56)	NS
Late (≥51 h)	388	301 (78)	173 (45)	77 (45)	NS
Total	845	666 (79)	366 (43)	194 (53)	NS
Natural mating	–	–	173	96 (55)	NS

Abbreviations: NS, not significant; S, significant.

^a^ Comparison between the expected ratio and the actual ratio obtained for respective trial.

^b^ In vitro fertilized embryos were classified as to “early” (≤47 h after insemination), “intermediate” (48‐50 h after insemination), and “late” (≥51 h after insemination) development based on the time of the third cleavage in culture.

In this study, the sex of total 1066 blastocysts derived from IVF was determined by PCR analysis. Approximately 58% (620/1066) of the embryos were male (*P* < .0001), whereas 53% (194/366) of fetuses derived from transfer of the fertilized embryos into recipient animals were male (*P* = .2502). The fetal sex ratio (55%; 96/173) derived from natural mating (*P* = .1486) was comparable to that of IVF and subsequent embryo transfer.

These results indicated that there might be a deviation for postimplantation development between the male and female embryos.

## 4 DISCUSSION

Our study indicated that male embryos tend to develop faster than female embryos during early stage of preimplantation in mice (Tables 1 and 2) and support a previous study.^2^, ^3^ Valdivia et al ^3^ showed that the sex ratio deviations observed at the preimplantation stage are maintained throughout the postimplantation development in mice. However, in the present study, sex ratio (% male) of 90% of the early‐developing embryos that exhibited third cleavage as early as 47 h after insemination were male. Moreover, early‐developing embryos produced 80% males after embryo transfer (Table 3). Furthermore, 59% of the total blastocysts and 53% of the total full‐term fetuses were male. The rates of live fetuses inclined to be more male embryos than female embryos, namely there might be a substantial loss of male embryos during implantation and/or postimplantation development after the embryo transfer.^12^ Therefore, the overall male:female sex ratio may be regulated toward 1:1 through postimplantation development. However, contradictory results have been reported about the sex ratio at the blastocyst stage in mice by Tan et al.^13^ They showed that 51% of blastocysts derived from IVF were male, and their sex ratio at birth was skewed due to an increased percentage of apoptosis and dysregulated expression of representative sex‐dimorphic genes. As the sperm penetration into the oocyte was completed within 1 hour after insemination in the IVF procedure carried out in the present study,^3^, ^7^, ^14^, ^15^ the time difference between the third cleavage in early‐ and late‐developing embryos was not due to the difference in the time of fertilization but primarily because of the rate of embryonic development after IVF.

A skewed sex ratio in favor of the male gender was reported in calves born through artificial insemination; however, there was no evidence indicating that the primary sex ratio in spermatozoa, both at thawing and after swim‐up, differed from the theoretical 1:1 ratio.^16^ Thus, difference between genders observed after artificial insemination might be due to the events occurring at or after fertilization, which could comprise impaired function of the X‐ or Y‐bearing spermatozoa with consequences on embryonic development. Although there were several reports that human IVF resulted in a skewed sex ratio at birth,^17^, ^18^, ^19^ clinical IVF for infertility treatment in humans might be affected by specific disruption of embryonic development or suboptimal environmental factors. The skewed sex ratio induced by environmental factors might occur through epigenetic mechanisms. Epidemiologic analyses based on large clinical population indicated higher rate of male birth through IVF,^17^, ^18^ which is in agreement with our results in mice. However, intracytoplasmic sperm injection (ICSI) resulted in a female‐biased sex ratio in humans.^12^, ^17^, ^19^ Therefore, ICSI might affect sex ratio through some epigenetic mechanisms.

Thus, in summary, our study indicated a tendency toward male gender in early‐developing mouse embryos fertilized in vitro. Examination of the developing embryos using a time‐lapse monitoring system would provide more and precise information with respect to male advantage in faster developing mouse embryos. Furthermore, male and female embryos seem to differ with respect to their chromosomal complement, transcriptome, proteome, and metabolome, and in their epigenetic process, including X‐chromosome inactivation, DNA methylation, and gene imprinting. Although the notable candidate epigenome change which induced faster developing embryos in male has not been reported, variations in some of these epigenetic mechanisms between male and female preimplantation embryos may provide a molecular basis for male advantage in faster developing embryos in mice.

## CONFLICT OF INTEREST

The authors declare that they have no conflicts of interest.

## HUMAN AND ANIMAL RIGHTS

This article does not describe any experiments involving human participants. All of the institutional and national guidelines for the care and use of laboratory animals were followed. The protocol for the research project was approved by a suitably constituted ethics committee.
